# When TADs go bad: chromatin structure and nuclear organisation in human disease

**DOI:** 10.12688/f1000research.10792.1

**Published:** 2017-03-24

**Authors:** Vera B Kaiser, Colin A Semple

**Affiliations:** 1 MRC Human Genetics Unit, MRC Institute of Genetics and Molecular Medicine, The University of Edinburgh, Western General Hospital, Crewe Road, Edinburgh, EH4 2XU, UK

**Keywords:** TADs, developmental disorders, chromatin organisation

## Abstract

Chromatin in the interphase nucleus is organised as a hierarchical series of structural domains, including self-interacting domains called topologically associating domains (TADs). This arrangement is thought to bring enhancers into closer physical proximity with their target genes, which often are located hundreds of kilobases away in linear genomic distance. TADs are demarcated by boundary regions bound by architectural proteins, such as CTCF and cohesin, although much remains to be discovered about the structure and function of these domains. Recent studies of TAD boundaries disrupted in engineered mouse models show that boundary mutations can recapitulate human developmental disorders as a result of aberrant promoter-enhancer interactions in the affected TADs. Similar boundary disruptions in certain cancers can result in oncogene overexpression, and CTCF binding sites at boundaries appear to be hyper-mutated across cancers. Further insights into chromatin organisation, in parallel with accumulating whole genome sequence data for disease cohorts, are likely to yield additional valuable insights into the roles of noncoding sequence variation in human disease.

## Introduction

The past decade has seen a series of revolutions in the fields of human chromatin structure and regulatory genomics, driven largely by novel, high-throughput sequencing-based assays and large consortium projects. Projects such as Encyclopedia of DNA Elements (ENCODE) ^1^ have produced large datasets delineating the fine-scale landscape of regulatory elements active in hundreds of cell types by using chromatin immunoprecipitation followed by sequencing (ChIP-seq), DNase I treatment of DNA followed by high-throughput sequencing to determine active regulatory sites (DNAse-seq), isolation of RNA followed by sequencing (RNA-seq), and other methods. These data have provided a “locus level” view of the regulatory protein binding events and modifications to chromatin occurring (generally over tens or hundreds of base pairs) at individual genes. Meanwhile, the development of chromatin conformation capture (3C) methods, most prominently high-throughput chromosome conformation capture (Hi-C), has provided a view of the higher-order folding and nuclear organisation of human chromosomes ^2^. These data reveal a landscape of physically interacting regions (typically separated by tens or hundreds of kilobases) along chromosomes and, at a larger scale, the existence of preferentially self-interacting chromatin domains, extending across hundreds of kilobases up to multi-megabase regions. However, there is no shortage of gaps in our present knowledge ^3^. The mechanistic relationships between the hierarchical structural layers of the epigenome, from locus-level features to higher-order structures and three-dimensional nuclear architecture, are still the subject of active research. We also have an incomplete picture of how genome functions (such as transcription, replication, and repair) are driven or constrained by these structures. Beyond this, there has been little evidence for any phenotypic consequences of the disruption of the interactions and domains emerging from Hi-C experiments. However, a number of recent studies have provided new insights into the consequences of pathogenic mutations acting to alter domain structures and compromise genome function.

## The rise of the topologically associating domains

The original 3C method ^4^ and its derivatives—4C, 5C, and Hi-C—are used to study *in vivo* contact frequencies between pairs of genomic sequences, revealing the physical arrangement of DNA in the nucleus. Hi-C was developed to assess patterns of interaction between all regions of a given size across the entire genome simultaneously ^5^. The 3C methods have continued to evolve ^2^, but Hi-C was the original high-throughput, genome-wide variant of 3C and still accounts for the majority of 3C datasets available. The earliest low-resolution Hi-C maps showed interactions between 1 Mb regions, confirming the existence of nuclear compartments corresponding to multi-megabase regions within the accessible and transcriptionally active “A” compartment and regions within the relatively closed and inactive “B” compartment ^5^. The highest impact of such datasets was undoubtedly the discovery of topologically associating domains (TADs), thought to represent regulatory domains, which contain many preferentially interacting subregions but very few interactions across their boundaries. TAD boundaries are often defined by relatively simple algorithms, assessing the directional (upstream versus downstream) preferences of interactions occurring along a chromosome and defining boundaries where these preferences change ^6^. TADs were reported in multiple species as structural entities up to around 1 Mb in size in mammals (and perhaps half this length in Drosophila) and enriched for interacting promoter-enhancer contacts ^6– 8^. These early studies noted the association of these domains with varying regional patterns of repressive and activating histone modifications across the genome, the enrichment of CTCF binding at boundaries, and correlations with other aspects of higher-order chromatin organisation, such as patterns of association with the nuclear lamina and replication timing domains ^6^. About 50% of TADs appear to be cell type invariant between embryonic stem cells (ESCs) and the cortex ^6^, and domains are often shared across species boundaries ^6, 9^. Later work showed differing features enriched at boundaries in different cell types, and certain enrichments (for example, features associated with active promoters, CTCF, and YY1 binding) were seen across all cell types ^10^. Although CTCF has been intensively studied as an insulator protein, it is not enriched or even detectable at all boundaries, and so the architecture(s) of TAD boundary regions and the mechanism(s) underlying their function remain elusive.

The highest-resolution Hi-C datasets have revealed finer-scale, recurrent pairwise interactions between regions, representing “chromatin loops”, and the base of each loop is formed by two presumably interacting anchor points. Many of these loop anchor points appear to be bound by CTCF and cohesin subunits, and a majority were found to contain convergently orientated CTCF binding motifs ^11^. Since around 60% of CTCF sites are constitutively bound ^12^, a substantial fraction of loops may be present across tissues. The same study ^11^ identified many TAD-like “contact domains” ranging from 40 Kb to 3 Mb in size (median length of 185 Kb), suggesting that a small fraction of boundaries were missed by previous studies with lower-resolution data. Closer investigation of Hi-C interaction matrices revealed that TADs appear to interact in clusters to form a hierarchy of nested domains, or “metaTADs”, at higher levels ^13^. This hierarchy can be modelled as a simple tree-like structure, and rearrangements of the tree link changes in nuclear organisation to transcriptional changes at many promoters during neuronal differentiation—from ESCs via neural progenitor cells to neurons. This tree-like model of organisation provides an intuitive bridge between smaller-scale regulatory domains such as TADs and the A and B nuclear compartments ^13^.

**Figure 1.**
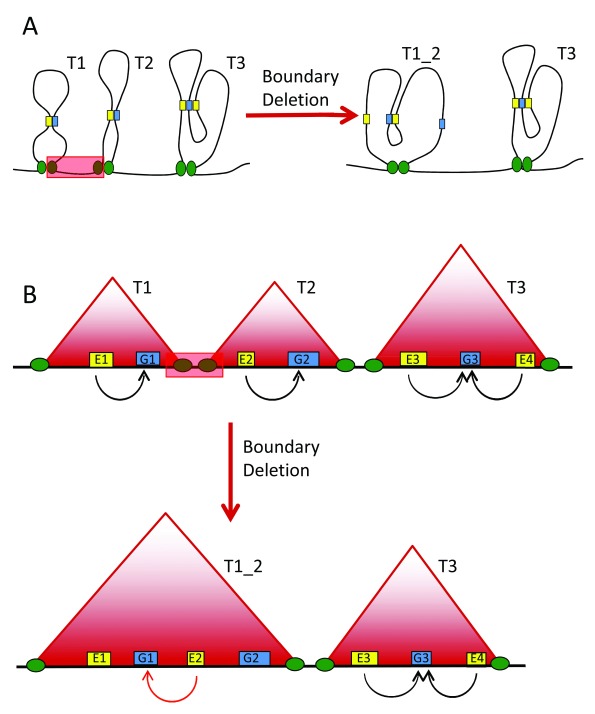
Schematic diagram of regulatory re-wiring following the deletion of a domain boundary. ( **A**) Interactions between enhancers and their target genes occur within chromatin domains. The deletion of a boundary region leads to novel gene-enhancer interactions between previously insulated elements; this process may lead to the spatial or temporal mis-expression of genes. ( **B**) The same scenario as in ( **A**) is drawn as represented by a high-throughput chromosome conformation capture (Hi-C) interaction map. Red triangles: topologically associating domains; yellow boxes: regulatory elements; blue boxes: target genes; green circles: insulator elements. Further examples of pathogenic genomic rearrangements, including insulator-spanning tandem duplications, are illustrated in 31.

A broad structural hierarchy has emerged, from the subgenic, locus-level features that can be linked by chromatin loops and constrained within TADs up to large nuclear compartment domains associated with patterns of transcription, replication, and nuclear localisation. Until recently, however, functional studies of domains have lagged because of the dearth of experimental approaches available to manipulate domains and assess the phenotypic effects ^3^, and some have questioned whether TAD organisation is a cause or consequence of genome function ^14^. As for other noncoding genomic sequences, the emphasis is shifting from the immediate biochemical roles of these new structural features to their phenotypic relevance and how their disruption impacts the fitness of real organisms ^15^. Here, we review the latest studies of dysregulation of gene expression due to changes in chromatin conformation ( Figure 1), focussing on developmental genetic disorders and cancer genomics. However, the unifying principle of mis-expression due to aberrant spatial organisation holds across somatic and germline mutations and is likely to extend to other phenotypes and complex traits.

## Chromatin domain lesions in development

Recent studies of particular mouse loci have implicated disrupted regulatory architecture in developmental disorders, and engineered mouse mutations have been seen to alter TAD boundaries and affect the expression of nearby genes ^16^. In a seminal study, Lupiáñez *et al*. ^17^ demonstrated the impact of TAD boundary disruption on gene expression in the developing limb bud. Three types of human limb malformations were shown to be associated with genomic rearrangements near the EHPA4 locus, including deletions, duplications, and an inversion affecting the TAD spanning the locus. Re-engineering the same rearrangements in mice, they found a general pattern of upregulation of genes in neighbouring TADs whose boundaries had been affected. Furthermore, these genes were now expressed in a spatiotemporal pattern that resembled endogenous Epha4 expression. Using 4C, they showed that genes in neighbouring TADs had acquired new interactions with the enhancer cluster of Epha4, a scenario named “enhancer adoption” ^18^. Interestingly, if the boundary regions were deleted but CTCF sites were maintained intact, no such re-wiring of interactions took place, emphasising the special importance of CTCF as a boundary element.

The extent of such disruptions in human developmental disease cases is largely unknown, but recent studies have shown that pathogenic human structural variants (SVs) overlap the boundary regions of TADs, including duplications at the SOX9 locus ^19^ and deletions at the MEF2C locus ^20^ in certain cases of developmental disorders. Furthermore, SVs disrupting an orthologous TAD boundary (found in human and mouse nuclear organisation) can give rise to the same developmental defect ^19^, suggesting a conserved functional role. A computational study assessed the broader importance of copy number variants (CNVs) affecting boundary elements on human developmental disorders ^21^. The phenotypes of 922 deletion cases recorded in the DECIPHER database ^22^ were related to the known, monogenic diseases associated with genes neighbouring the deletions. This information was used to nominate deletions likely to affect TAD boundaries and result in enhancer adoption. The results suggest that up to about 12% of deletions in the DECIPHER database resulted in enhancer adoption, based on the tissue-specific activity of a given enhancer and the tissue that was affected in the disease phenotype of the patient who carried the deletion.

## Chromatin domain lesions in cancer

There have been several reports of disruptions of particular TAD boundaries in modest numbers of neuroblastoma ^23, 24^, medulloblastoma ^25^, and leukaemia ^26, 27^ samples, consistent with the hypothesis that TAD-disrupting SVs may act as oncogenic “driver” mutations under selection in tumour cells ^28^. Proposed models for such phenomena include (a) deletions of boundaries to allow unusual promoter-enhancer contacts and (b) inversions that span boundaries altering the contents of neighbouring TADs; both mechanisms are thought to give rise to aberrant expression of resident genes. It also seems that boundary integrity can be compromised by hyper-methylation. Flavahan *et al*. ^29^ showed that gain-of-function mutations in the *IDH* gene cause hyper-methylation of CpG sites as well as the GC-rich CTCF binding motif; this reduces CTCF binding at a subset of CTCF sites in mutant glioma cells. Genes most upregulated in *IDH* mutants as a result of this hyper-methylation included several known oncogenes, such as *PDGFRA*. Furthermore, CRISPR disruption of a neighbouring CTCF binding site at a TAD boundary in wild-type tumours leads to the upregulation of *PDGFRA*, providing mutant cells with a growth advantage and enhancing proliferation. Intriguingly, the *PDGFRA* domain boundary affected by aberrant methylation in tumours can be re-established by treating mutant glioma cells with a de-methylation agent, leading to the downregulation of *PDGFRA*.

Hnisz *et al*. ^27^ investigated the impact of domain boundary deletions on oncogene activation in T-cell acute lymphoblastic leukaemia (T-ALL). Using ChIA-PET (chromatin interaction analysis by paired-end tag sequencing) against cohesin sites, they identified around 9,000 CTCF-CTCF interactions that were shared across cell lines, demarcating active loop anchor points for “constitutive neighbourhoods”. Most genes implicated in T-ALL pathogenesis were located inside those loops. Next, they investigated the overlaps between cancer deletions (<500 Kb) in a set of T-ALL whole genome sequences and these neighbourhood boundaries. Six boundaries delineating neighbourhoods containing T-ALL pathogenesis genes were found to be recurrently deleted across tumours at unexpectedly frequent levels. Hnisz *et al*. ^27^ then showed that the CRISPR-engineered deletion of boundaries near two known oncogenes ( *TAL1* and *LMO2*) leads to the activation of these genes in human embryonic kidney cells, which otherwise do not express these genes. They also demonstrated changes in conformation at these boundary regions using 5C, with the intensity of contacts increasing across the boundaries following the deletion.

A larger recent study used matched expression and somatic variation data for 7,416 cancer genomes across 26 tumour types from The Cancer Genome Atlas to systematically identify somatic copy number alterations (SCNAs) likely to mediate gene dysregulation ^30^. They found that recurrent SCNAs deleting a boundary of the TAD containing the *IRS4* gene were associated with a marked increase in *IRS4* expression in 32 samples from three different tumour types. These deletions were carefully distinguished from samples carrying focal amplifications leading to *IRS4* overexpression, and the tumorigenic effects of higher *IRS4* expression were validated *in vivo* using mouse models. Similar to ^19^, the authors also describe duplications mediating the formation of a new domain and driving overexpression of *IGF2* in 20 colorectal cancer (CRC) tumours. Single copy tandem duplications encompassing an *IGF2* TAD boundary and an enhancer in a neighbouring TAD were found to alter conformation and activate the enhancer in CRC cell lines, leading to overexpression of *IGF2*, a gene previously implicated in CRC progression. Interestingly, all duplications involving this locus were tandem duplications—rather than dispersed or inverted duplications—suggesting that the resulting head-to-tail orientation of the enhancer and *IGF2* may be crucial for the gene’s upregulation ^31^. Overall, these data suggest that enhancer adoption by oncogenes following domain boundary lesions is not a rare process, occurring at rates comparable to those of recurrent in-frame gene fusions.

## Disruption of CTCF sites in cancer

CTCF hemizygous knockout mice are prone to developing cancer in a wide range of tissues ^32^, consistent with oncogene activation following the rearrangement of nuclear architecture. Several recent studies have also observed unexpected excesses of somatic mutations at CTCF binding sites across tumour types, including CRC ^33^, leukaemia ^27^, and a variety of other cancer types ^34^. Characteristic mutational profiles were observed at positions within the CTCF binding motif, and a striking spike in mutation was seen at a central, well-conserved nucleotide, together with elevated mutation rates at sites immediately flanking the motif ^33, 34^. Surprisingly, these unusual patterns can be explained by selectively neutral biases in mutation rates ^34^ and are broadly consistent with an underlying mutational mechanism involving the interference of DNA binding proteins with the replication machinery, causing elevated mutational burden at active regulatory sites ^35^. The highest mutational loads were seen at constitutively active CTCF binding sites with roles in chromatin loops and TAD domain boundaries and were predicted to compromise CTCF binding and nuclear architecture ^34^. These phenomena therefore appear to provide a deterministic mutation-driven process, expected to lead to altered chromatin architecture and gene dysregulation in many cancers, without invoking more complex hypotheses involving similar selective pressures across many sites and many different tumour types.

## Future challenges

The future will undoubtedly provide many more insights into the disruptions of chromatin and nuclear organisation underlying disease. Continuing advances in high-throughput sequencing and computational analyses are improving the resolution of Hi-C maps and allow greater precision in defining domain boundaries and other structures. Global analyses of structural disruptions in disease have generally been limited to publicly available Hi-C data, which usually are not well matched to the tissue of interest. Similar caveats apply to the available ChIP-seq data for chromatin features such as CTCF binding. With more cell- and tissue-specific Hi-C and ChIP-seq data available, many more disease-relevant disrupted interactions are likely to be found. This will also help to better distinguish “passenger” disruptions of TAD boundaries from causative “driver” mutations responsible for oncogene dysregulation. Current cancer whole genome sequencing (WGS) datasets are also inadequate for these purposes. Previous studies have examined mutational spectra at thousands of CTCF binding sites across the genome ^33, 34^ and are underpowered to discover individual CTCF sites subject to recurrent mutation. Similarly, most WGS data for tumours lack matched RNA-seq data to study the effects of site disruption on the patterns of expression of neighbouring genes.

In addition to improvements in the data available, there are substantial challenges ahead in WGS data analysis. Currently available algorithms for the detection of CNVs (that is, deletions and duplications) are generally used in combinations to generate consensus predictions since no single algorithm is considered sufficiently accurate when used alone ^36^. More complex SVs, such as inversions and translocations, are even less accurately predicted, and compound SVs (involving different overlapping or nested SVs) are beyond our reach entirely. This point is highly pertinent to cancer genomics since catastrophic rearrangements of entire chromosomes are an increasingly common observation in many cancers ^37^, and SVs appear to be the main factors driving tumorigenesis in some cases ^38^. Given the challenges of reconstructing SVs from standard (short read) sequencing data, it is likely that accurate resolution of all SVs present in a genome will be dependent upon novel sequencing technologies generating much longer sequence reads (for example, ^39^).

As discussed above, our knowledge of the hierarchical strata and functional inter-relationships of higher-order chromatin structure is still far from complete. Although TADs have been widely studied, we still lack detailed models of how they affect transcription. Sharing a location in the same TAD is evidently not sufficient to allow enhancer-promoter interactions to take place, and we do not understand how enhancers find their target genes within TADs. Some data suggest that a particular spacing may be needed to create a loop that successfully brings such elements together, and it is known that increasing the distance between an enhancer and a target promoter can lead to downregulation ^40^. We also know very little about the precise physical location and composition of the protein complexes bound to domain boundaries, how the insulating effect of boundaries is achieved, and what constitutes the minimal requirements for boundary function. Higher-resolution maps derived from Hi-C or related methods will be necessary to explore the detailed features of boundary regions. The fact that some boundaries appear to lack CTCF binding sites, that many TAD boundaries change during cellular differentiation ^13^, and that boundary compositions vary between cell types ^10^ suggests that boundaries may vary in structure and function. The better-studied boundary components, CTCF and cohesin, appear to have distinct functions in domain formation, in that CTCF works to separate neighbouring TADs and cohesin promotes intra-TAD interactions ^41^, but even these proteins remain the subject of ongoing research. A comprehensive picture of boundary architecture and function holds the promise of better understanding, and perhaps correcting, regulatory domain disruptions in disease.

## Abbreviations

3C, chromatin conformation capture; ChIP-seq, chromatin immunoprecipitation followed by sequencing; ChIA-PET, chromatin interaction analysis by paired-end tag sequencing; CRISPR, the CRISPR-Cas9 molecular editing system allows alterations to DNA at specific locations in the genome; Hi-C, high-throughput chromosome conformation capture; RNA-seq, isolation of RNA followed by sequencing; TAD, topologically associating domain.
